# Molecular epidemiology and patient outcome of carbapenem-resistant *Enterobacterales, Pseudomonas aeruginosa* and *Acinetobacter baumannii* in Japan: a multicenter study from MultiDrug-Resistant organisms clinical research network

**DOI:** 10.1093/jacamr/dlaf027

**Published:** 2025-03-06

**Authors:** Sho Saito, Aki Sakurai, Yasufumi Matsumura, Kohei Uemura, Ryota Hase, Hideaki Kato, Naoya Itoh, Takehiro Hashimoto, Takashi Matono, Jiefu Yu, Kayoko Hayakawa, Masahiro Suzuki, Shoki Izumi, Tetsuya Suzuki, Mari Kurokawa, Koh Shinohara, Keiichiro Mori, Yasunobu Endo, Haruki Mito, Kayoko Sano, Tomo Matsunaga, Nana Akazawa, Kazufumi Hiramatsu, Yusuke Asai, Shinya Tsuzuki, David van Duin, Norio Ohmagari, Yohei Doi

**Affiliations:** Disease Control and Prevention Center, National Center for Global Health and Medicine, 1-21-1 Toyama, Shinjuku-ku 162-8655, Tokyo, Japan; Disease Control and Prevention Center, National Center for Global Health and Medicine, 1-21-1 Toyama, Shinjuku-ku 162-8655, Tokyo, Japan; Departments of Microbiology and Infectious Diseases, Fujita Health University School of Medicine, Aichi, Japan; Department of Clinical Laboratory Medicine, Kyoto University Graduate School of Medicine, Kyoto, Japan; Biostatistics and Bioinformatics Course, The University of Tokyo, Tokyo, Japan; Department of Infectious Diseases, Japanese Red Cross Narita Hospital, Chiba, Japan; Infection Prevention and Control Department, Yokohama City University Hospital, Kanagawa, Japan; Division of Infectious Diseases, Aichi Cancer Center Hospital, Aichi, Japan; Department of Infectious Diseases, Graduate School of Medical Sciences, Nagoya City University, Aichi, Japan; Department of Infectious Diseases, Nagoya City University East Medical Center, Aichi, Japan; Hospital Infection Control Center, Oita University Hospital, Oita, Japan; Department of Infectious Diseases, Aso Iizuka Hospital, Fukuoka, Japan; Division of Infectious Disease and Hospital Epidemiology, Saga University Hospital, Saga, Japan; AMR Clinical Reference Center, National Center for Global Health and Medicine, Tokyo, Japan; Disease Control and Prevention Center, National Center for Global Health and Medicine, 1-21-1 Toyama, Shinjuku-ku 162-8655, Tokyo, Japan; AMR Clinical Reference Center, National Center for Global Health and Medicine, Tokyo, Japan; Departments of Microbiology and Infectious Diseases, Fujita Health University School of Medicine, Aichi, Japan; Biostatistics and Bioinformatics Course, The University of Tokyo, Tokyo, Japan; Disease Control and Prevention Center, National Center for Global Health and Medicine, 1-21-1 Toyama, Shinjuku-ku 162-8655, Tokyo, Japan; Departments of Microbiology and Infectious Diseases, Fujita Health University School of Medicine, Aichi, Japan; Department of Clinical Laboratory Medicine, Kyoto University Graduate School of Medicine, Kyoto, Japan; Department of Clinical Laboratory, Kyoto University Hospital, Kyoto, Japan; Department of Laboratory Medicine, Japanese Red Cross Narita Hospital, Chiba, Japan; Department of Infectious Diseases, Japanese Red Cross Narita Hospital, Chiba, Japan; Clinical Laboratory Department, Yokohama City University Hospital, Kanagawa, Japan; Clinical Laboratory Department, Yokohama City University Hospital, Kanagawa, Japan; Division of Infectious Diseases, Aichi Cancer Center Hospital, Aichi, Japan; Department of Infectious Diseases, Graduate School of Medical Sciences, Nagoya City University, Aichi, Japan; Department of Infectious Diseases, Nagoya City University East Medical Center, Aichi, Japan; Hospital Infection Control Center, Oita University Hospital, Oita, Japan; AMR Clinical Reference Center, National Center for Global Health and Medicine, Tokyo, Japan; AMR Clinical Reference Center, National Center for Global Health and Medicine, Tokyo, Japan; Division of Infectious Diseases, University of North Carolina School of Medicine, Chapel Hill, NC, USA; Disease Control and Prevention Center, National Center for Global Health and Medicine, 1-21-1 Toyama, Shinjuku-ku 162-8655, Tokyo, Japan; AMR Clinical Reference Center, National Center for Global Health and Medicine, Tokyo, Japan; Departments of Microbiology and Infectious Diseases, Fujita Health University School of Medicine, Aichi, Japan; Division of Infectious Diseases, University of Pittsburgh School of Medicine, Pittsburgh, PA, USA

## Abstract

**Background and objectives:**

Carbapenem-resistant Gram-negative bacilli (CRGNB), especially *Enterobacterales, Pseudomonas aeruginosa* and *Acinetobacter baumannii*, are critical pathogens associated with excess morbidity and mortality. To elucidate their molecular epidemiology and clinical outcomes in Japan, patients with CRGNB were enrolled in the MDR organisms clinical research network (MDRnet) consisting of eight tertiary care facilities.

**Methods:**

Between 2019 and 2022, 246 unique patients with carbapenem-resistant *Enterobacterales* (CRE), carbapenem-resistant *P. aeruginosa* (CRPA) and carbapenem-resistant *A. baumannii* (CRAB) isolates were prospectively enrolled.

**Results:**

A total of 246 isolates were collected from 246 patients, including 78 (31.7%) CRE, 167 (67.9%) CRPA and 1 (0.4%) CRAB. For CRE, 74.4% of the isolates carried carbapenemase genes with predominance of *bla*_IMP_ (64.1%). Only 2.4% of CRPA had carbapenemase genes, which was lower than CRE. Among the infected patients, 20.0% and 12.5% died of CRE and CRPA within 30 days, respectively. In patients with CRE, the mortality rate within 30 days for those without carbapenemase-producing *Enterobacterales* (CPE) was higher compared with those with CPE (22.2% compared with 18.8%).

**Conclusions:**

Our study highlights the unique molecular epidemiology and clinical outcomes of CRGNB in Japan.

## Introduction

Carbapenem-resistant Gram-negative bacilli (CRGNB) are increasingly implicated in difficult-to-treat infections and are a serious global health concern. The World Health Organization has identified carbapenem-resistant *Enterobacterales* (CRE), carbapenem-resistant *Pseudomonas aeruginosa* (CRPA) and carbapenem-resistant *Acinetobacter baumannii* (CRAB) as priority pathogens in urgent need of research and development of new therapeutics.^1^

Carbapenemase-producing *Enterobacterales* (CPE) are particularly problematic among CREs. An overall mortality rate of 41% is reported for *Klebsiella pneumoniae* carbapenemase (KPC)–producing *Enterobacterales* infections, which are common in the Americas and China.^2,3^ Infections due to New Delhi metallo-β-lactamase (NDM) –producing *Enterobacterales* are frequently encountered in South Asia and carry a mortality rate of 23%–25%.^4^ Infections caused by *Enterobacterales* producing imipenemase (IMP) –type carbapenemases are associated with mortality rates of 16%–27%.^5,6^ However, strains producing IMP-type carbapenemases are limited in their geographic distribution, and reports on their molecular epidemiology and clinical outcomes are limited.^7^

CRPA infections are also associated with high mortality, with 30 day mortality rates of around 30% for bloodstream infections.^8–10^ The proportion of CRPA isolates producing carbapenemase varies widely by region, ranging from 2% in the USA to 32% in China and 69% in South and Central America.^10^ CRAB is a major problem worldwide, with particularly high detection rates reported in the Asia Pacific region.^11^ Most CRAB isolates produce carbapenemase, and the mortality rate of bloodstream infections caused by CRAB is as high as 50%.^12^

The distribution of genes encoding carbapenemases in Japan differs from that in other countries, with *bla*_IMP_ accounting for the majority.^6,13^ In addition, the reported prevalence of CRAB is extremely low.^14^ However, how the unique epidemiology impacts outcomes of patients affected by CRE, CRPA and CRAB needs to be better understood. This study aimed to elucidate the molecular epidemiology and clinical outcomes of patients with CRE, CRPA and CRAB in hospitals across Japan and to compare the molecular epidemiology and clinical outcomes of those with CRE and CRPA.

## Materials and methods

### Study design and patients

We prospectively enrolled hospitalized patients from whom CRGNB was cultured from any clinical specimen type from April 2019 to March 2022 in the MDR organisms clinical research network (MDRnet) consisting of eight tertiary care facilities in Japan. We screened all CRGNB reported at the participating hospitals and included all unique hospitalized patients with CRE, CRPA and CRAB. The isolates were centrally retested by the disk diffusion method and whole-genome sequencing. Cases with confirmed carbapenem-resistant or carbapenemase gene-positive isolates were included. We defined isolates with resistance to meropenem as CRE (zone diameter ≤22 mm) and isolates with resistance to meropenem or imipenem as CRPA (zone diameter ≤18 mm) and CRAB (zone diameter ≤17 mm for meropenem and ≤21 mm for imipenem) based on the breakpoints defined by the CLSI.^15^ Since certain species, such as *Morganella morganii* and *Proteus mirabilis*, are intrinsically resistant to imipenem, only meropenem was used in the definition of CRE. In principle, all analyses were based on the first isolate during index hospitalization. However, if the first isolate represented colonization and the patient subsequently developed an infection, we used the isolate with which the infection was ascertained. If two or more isolates of the same species were detected on the same day, the isolate with a lower identification number was used for analysis. Specimens collected for active screening were excluded from the study.

### Microbiological investigation

The antimicrobial susceptibilities of the isolates were centrally determined by the broth microdilution method. The MICs of antimicrobial agents were obtained using customized 96-well broth microdilution panels (Eiken Chemical Co., Tokyo, Japan) in accordance with the manufacturer’s instructions. The results were interpreted according to the CLSI criteria (CLSI M100, 31st Ed). For tigecycline, an MIC of >0.5 mg/L was used to define resistance based on the European Committee on Antimicrobial Susceptibility Testing criteria. The percentages of MDR, XDR, and difficult-to-treat-resistant (DTR) were calculated for each isolate, as defined previously.^16,17^ For *Enterobacterales*, due to the limited number of antimicrobial agents tested for susceptibility, the isolates were classified as MDR or XDR if they were resistant to at least 3 or 10 antimicrobial agents, respectively, out of 12 categories (aminoglycoside, antipseudomonal penicillin + β-lactamase inhibitor, carbapenem, expanded-spectrum cephalosporin, fluoroquinolone, folate pathway inhibitor, glycylcycline, monobactam, penicillin/β-lactamase inhibitor, phosphonic acid, polymyxin and tetracycline). For *P. aeruginosa*, the isolates were classified as MDR or XDR if they were resistant to at least 3 or 5 agents, respectively, out of 7 categories (aminoglycoside, antipseudomonal carbapenem, antipseudomonal cephalosporin, antipseudomonal fluoroquinolone, antipseudomonal penicillin/β-lactamase inhibitor, monobactam and polymyxin).

### Definitions of infection

A patient with a positive culture for CRE, CRPA and CRAB was considered to have an infection if they were isolated from the blood or other normally sterile sources such as ascites. We used the American Heart Association diagnostic definitions for infective endocarditis and the Centers for Disease Control and Prevention definitions for central line–associated bloodstream infection, respiratory tract infection, urinary tract infection and surgical site infection.^18–22^ Patients with cultures from sites other than those listed above were only considered infected if they had evidence of systemic inflammation on the day of the positive culture. Systemic inflammation was defined as elevated or low white blood cell count (>10 000 or <4000 cells/mm^3^, respectively) and/or an abnormal body temperature (>37.5°C or <35.5°C).^23^ Isolates were considered to represent colonization if patients did not meet any of the above criteria.

### Data collection

Clinical data were collected from the medical records and entered into a RedCap database. The data for the following parameters were collected from electronic medical records: demographics (age and sex), the origin of admission for the index hospitalization, comorbidities (diabetes mellitus, cardiovascular disease, pulmonary disease, renal disease, hepatic disease, central nervous system disease, malignancy and the overall number of comorbid conditions, including Charlson comorbidity scores), immunosuppression, overseas travel history within 3 months, Pitt bacteraemia score at collection of culture specimen (all patients), patient location at time of culture, culture sites, infection sites (clinical focus of infection) and outcomes (including 30 day mortality and length of hospital stay after the index culture).^24,25^ If the comorbidity included dementia and disorientation, the mental status was coded as ‘disoriented’ only for calculating the Pitt bacteraemia score. An immunosuppressed state was defined as a history of steroid use within 30 days and a history of chemotherapy, use of biological agents or immunosuppressants within 3 months of the date of the index culture. The definition of steroid administration did not include cut-off value for the dosage, and the route of administration was either oral or intravenous. The Institutional Review Board of the National Center for Global Health and Medicine (NCGM-S-003163) approved this study at the principal investigator site and subsequently approved it at each participating site, granting the opt-out process for informed consent.

### Molecular detection of species and carbapenemases

Genomic DNA was extracted from isolates using the DNeasy blood and tissue kit (Qiagen, Tokyo, Japan) or the MagNA Pure 96 DNA and Viral NA Small Volume extraction kit per the manufacturer’s instructions. Whole-genome sequencing was performed with NextSeq 2000 or NextSeq1000 (Illumina, Inc., San Diego, CA, USA), using 2 × 150 bp paired-end reads. *De novo* genome assembly was performed with SPAdes. Species identification was conducted by calculating the orthologous average nucleotide identity (OrthoANI) value between the genome sequence obtained from study isolates and the reference genome for each bacterial species, which is available in Table S1 (available as Supplementary data at *JAC-AMR* Online). An OrthoANI cut-off value of 95% was used for species delineation. Sequence types were determined *in silico* according to the PubMLST database (https://pubmlst.org/) or the Institute Pasteur MLST database (http://bigsdb.pasteur.fr/), utilizing the software MLST v2.0.9 of the Center for Genomic Epidemiology (http://genomicepidemiology.org/). Acquired carbapenemase genes were identified using AMRFinderPlus.^26^

### Statistical analysis

Continuous variables were described as median and IQR and categorical variables as number of cases and percentages. *P* values were calculated to compare demographic characteristics, clinical outcomes, types of infections and susceptibility based on Wilcoxon’s rank sum test for continuous variables and Fisher’s exact test for categorical variables. Thirty-day mortality rates, total length of stay and length of stay after culture collection were also analysed as time-to-event data, which meant time from the index positive culture to death after adjusting patients’ background information by inverse-probability treatment weighting method using propensity score. The propensity score was calculated by logistic regression analysis including age, sex, Charlson comorbidity index score, Pitt bacteraemia score, evidence of a bloodstream infection and evidence of respiratory infections. Standardized mean difference >0.2 was interpreted as a meaningful imbalance. We used Cox regression analysis for 30 day mortality rate and quasi-Poisson regression for length of stays. All statistical analyses were performed using SAS software version 9.4 (SAS Institute, Cary, NC, USA), R version 4.3.2 (R Core Team, 2018; R Foundation for Statistical Computing, Vienna, Austria) and SPSS version 28.0.1.1 (IBM Corporation, Armonkm, NY, USA).

## Results

### Patients

A total of 246 patients with CRE, CRPA and CRAB were enrolled at eight tertiary care facilities in Japan between 2019 and 2022. Patients were generally advanced in age (median age 71 years; IQR, 59–78) and had various comorbidities, including a history of diabetes (25.2%), localized tumour (20.7%) or cerebrovascular disease (15.0%), with a median Charlson comorbidity score of 2 (IQR, 1–3) (Table 1). Patients with CRPA had significantly higher connective tissue disease rates and were more likely to be on glucocorticoid therapy than those with CRE (*P* = 0.001 and 0.007, respectively). In addition, data on neutrophil counts were collected for 209 patients, and 4 patients had neutropenia (<500 cells/mm^3^) (CRE 1 patient and CRPA 3 patients).

**Table 1.** Demographic characteristics and outcomes of patients with CRE, CRPA and CRAB

	All (*n* = 246)	CREs (*n* = 78)	CRPAs (*n* = 167)					*P* value^a^ All CREs versus all CRPAs	*P* value CRE infection versus CRPA infection	*P* value CRE colonization versus CRPA colonization
		All CREs (*n* = 78)	CRE infection (*n* = 25)	CRE colonization (*n* = 53)	All CRPAs (*n* = 167)	CRPA infection (*n* = 96)	CRPA colonization (*n* = 71)			
Age, median (IQR) (year)	71 (59–78)	70 (64–79)	67 (60–77)	71 (64–82)	71 (59–78)	72 (58–78)	71 (59–78)	0.977	0.463	0.664
Sex (male)	146 (59.3%)	51 (65.4%)	19 (76.0%)	32 (60.4%)	95 (56.9%)	56 (58.3%)	39 (54.9%)	0.213	0.164	0.585
From								0.514	0.131	0.313
Home	180 (73.2%)	55 (70.5%)	24 (96.0%)	31 (58.5%)	125 (74.9%)	74 (77.1%)	51 (71.8%)			
Another hospital	43 (17.5%)	15 (19.2%)	1 (4.0%)	14 (26.4%)	27 (16.2%)	14 (14.6%)	13 (18.3%)			
Nursing home	22 (8.9%)	7 (9.0%)	0 (0%)	7 (13.2%)	15 (9.0%)	8 (8.3%)	7 (9.9%)			
Birth in hospital	1 (0.4%)	1 (1.3%)	0 (0%)	1 (1.9%)	0 (0%)	0 (0%)	0 (0%)			
Acute and chronic conditions on admission										
Myocardial infarction	13 (5.3%)	3 (3.8%)	0 (0%)	3 (5.7%)	10 (6.0%)	7 (7.3%)	3 (4.2%)	0.760	0.343	1.000
Congestive heart failure	22 (8.9%)	10 (12.8%)	5 (20.0%)	5 (9.4%)	12 (7.2%)	10 (10.4%)	2 (2.8%)	0.157	0.303	0.136
Peripheral vascular disease	13 (5.3%)	3 (3.8%)	2 (8.0%)	1 (1.9%)	10 (6.0%)	3 (3.1%)	7 (9.9%)	0.760	0.275	0.136
Cerebrovascular disease	37 (15.0%)	14 (17.9%)	5 (20.0%)	9 (17.0%)	23 (13.8%)	14 (14.6%)	9 (12.7%)	0.445	0.541	0.608
Dementia	22 (8.9%)	7 (9.0%)	1 (4.0%)	6 (11.3%)	15 (9.0%)	5 (5.2%)	10 (14.1%)	1.000	1.000	0.789
Chronic obstructive pulmonary disease	8 (3.3%)	1 (1.3%)	0 (0%)	1 (1.9%)	7 (4.2%)	3 (3.1%)	4 (5.6%)	0.442	1.000	0.392
Connective tissue disease	24 (9.8%)	1 (1.3%)	0 (0%)	1 (1.9%)	23 (13.8%)	11 (11.5%)	12 (16.9%)	**0.001**	0.118	**0**.**007**
Peptic ulcer disease	1 (0.4%)	0 (0%)	0 (0%)	0 (0%)	1 (0.6%)	0 (0%)	1 (1.4%)	1.000	NA	1.000
Diabetes mellitus	62 (25.2%)	20 (25.6%)	8 (32.0%)	12 (22.6%)	42 (25.1%)	24 (25.0%)	18 (25.4%)	1.000	0.459	0.833
Any liver disease	14 (5.7%)	5 (6.4%)	3 (12.0%)	2 (3.9%)	9 (5.4%)	7 (7.3%)	2 (2.8%)	0.772	0.429	1.000
Any renal diseases	21 (8.5%)	7 (9.0%)	2 (8.0%)	5 (9.4%)	14 (8.4%)	10 (10.4%)	4 (5.6%)	1.000	1.000	0.495
Haemiplegia	10 (4.1%)	2 (2.6%)	2 (8.0%)	0 (0%)	8 (4.8%)	3 (3.1%)	5 (7.0%)	0.510	0.275	0.071
Leukaemia	5 (2.0%)	1 (1.3%)	1 (4.0%)	0 (0%)	4 (2.4%)	3 (3.1%)	1 (1.4%)	1.000	1.000	1.000
Lymphoma	3 (1.2%)	0 (0%)	0 (0%)	0 (0%)	3 (1.8%)	2 (2.1%)	1 (1.4%)	0.553	1.000	1.000
Localized solid tumor	51 (20.7%)	13 (16.7%)	3 (12.0%)	10 (18.9%)	37 (22.2%)	22 (22.9%)	15 (21.1%)	0.396	0.280	0.824
Metastatic solid tumor	20 (8.1%)	4 (5.1%)	2 (8.0%)	2 (3.8%)	16 (9.6%)	11 (11.5%)	5 (7.0%)	0.319	1.000	0.698
Charlson’s combined condition score, median (IQR)	2 (1–3)	1 (0–3)	2 (1–3)	1 (0–3)	2 (1–3)	2 (1–4)	2 (0–3)	0.037	0.546	0.100
Immunosuppressive state										
Glucocorticoid therapy	62 (25.2%)	11 (14.1%)	4 (16.0%)	7 (13.2%)	50 (29.9%)	30 (31.3%)	20 (28.2%)	**0**.**007**	0.210	0.051
Antineoplastic chemotherapy	12 (4.9%)	3 (3.8%)	1 (4.0%)	2 (3.8%)	8 (4.8%)	6 (6.3%)	2 (2.8%)	1.000	1.000	1.000
Biological agents	5 (2.0%)	3 (3.8%)	1 (4.0%)	2 (3.8%)	2 (1.2%)	0 (0%)	2 (2.8%)	0.330	0.207	1.000
Immunosuppressant	19 (7.7%)	3 (3.8%)	1 (4.0%)	2 (3.8%)	15 (9.0%)	12 (12.5%)	3 (4.2%)	0.193	0.299	1.000
Solid organ transplantation	10 (4.1%)	1 (1.3%)	1 (4.0%)	0 (0%)	8 (4.8%)	6 (6.3%)	2 (2.8%)	0.279	1.000	0.507
Haematopoietic stem cell transplantation	2 (0.8%)	0 (0%)	0 (0%)	0 (0%)	2 (1.2%)	1 (1.0%)	1 (1.4%)	1.000	1.000	1.000
Overseas travel history in the past 3 months								**<0**.**001**	0.022	**0**.**001**
Yes	6 (2.4%)	6 (7.7%)	0 (0%)	6 (11.3%)	0 (0%)	0 (0%)	0 (0%)			
No	150 (61.0%)	56 (71.8%)	20 (80.0%)	36 (67.9%)	93 (55.7%)	52 (54.2%)	41 (57.7%)			
Unknown	90 (36.6%)	16 (20.5%)	5 (20.0%)	11 (20.8%)	74 (44.3%)	44 (45.8%)	30 (42.3%)			
Location at time of culture collection								0.326	0.745	0.459
Emergency department	14 (5.7%)	2 (2.6%)	1 (4.0%)	1 (1.9%)	12 (7.2%)	8 (8.3%)	4 (5.6%)			
ICU	23 (9.3%)	9 (11.5%)	4 (16.0%)	5 (9.4%)	14 (8.4%)	10 (10.4%)	4 (5.6%)			
Ward	204 (82.9%)	67 (85.9%)	20 (80.0%)	47 (88.7%)	136 (81.4%)	73 (76.0%)	63 (88.7%)			
Pitt bacteraemia score, median (IQR)	1 (0–3)	1 (0–4)	0 (0–3)	2 (0–4)	1 (0–3)	1 (0–3)	1 (0–2)	0.435	0.513	0.172
Duration from hospitalization to isolation^a^								0.737	0.447	0.545
The same day	41 (16.7%)	11 (14.1%)	2 (8.0%)	9 (17.0%)	30 (18.0%)	17 (17.7%)	13 (18.3%)			
On Days 1–5	33 (13.4%)	10 (12.8%)	4 (16.0%)	6 (11.3%)	23 (13.8%)	10 (10.4%)	13 (18.3%)			
After Day 6	172 (69.9%)	57 (73.1%)	19 (76.0%)	38 (71.7%)	114 (68.3%)	69 (71.9%)	45 (63.4%)			
Culture sites										
Blood	22 (8.9%)	6 (7.7%)	6 (24.0%)	0 (0%)	16 (9.6%)	16 (16.7%)	0 (0%)	0.811	0.394	NA
Respiratory	97 (39.4%)	24 (30.8%)	6 (24.0%)	18 (34.0%)	73 (43.7%)	35 (36.5%)	38 (53.5%)	0.068	0.343	0.044
Urine	59 (24.0%)	22 (28.2%)	5 (20.0%)	17 (32.1%)	37 (22.2%)	20 (20.8%)	17 (23.9%)	0.337	1.000	0.416
Wound	18 (7.3%)	3 (3.8%)	2 (8.0%)	1 (1.9%)	15 (9.0%)	9 (9.4%)	6 (8.5%)	0.193	1.000	0.237
Stool	12 (4.9%)	12 (15.4%)	0 (0%)	12 (22.6%)	0 (0%)	0 (0%)	0 (0%)	**<0**.**001**	NA	**<0**.**001**
Other	45 (18.3%)	13 (16.7%)	7 (28.0%)	6 (11.3%)	31 (18.6%)	20 (20.8%)	11 (15.5%)	0.858	0.431	0.603
Type of infection^b^										
Bloodstream infection	22 (8.9%)	6 (7.7%)	6 (24.0%)	NA	16 (9.6%)	16 (16.7%)	NA	0.811	0.394	NA
Respiratory tract infection	40 (16.3%)	6 (7.7%)	6 (24.0%)	NA	34 (20.4%)	34 (35.4%)	NA	**0**.**015**	0.345	NA
Urinary tract infection	28 (11.4%)	6 (7.7%)	6 (24.0%)	NA	22 (13.2%)	22 (22.9%)	NA	0.282	1.000	NA
Surgical site infection	14 (5.7%)	3 (3.8%)	3 (12.0%)	NA	11 (6.6%)	11 (11.5%)	NA	0.558	1.000	NA
Others	28 (11.4%)	6 (7.7%)	6 (24.0%)	NA	22 (13.2%)	22 (22.9%)	NA	0.282	1.000	NA
Carbapenemase	63 (25.6%)	58 (74.4%)	16 (64.0%)	42 (79.2%)	4 (2.4%)	0 (0%)	4 (5.6%)	**<0**.**001**	**<0**.**001**	**<0**.**001**
IMP	53 (21.5%)	50 (64.1%)	16 (64.0%)	34 (64.2%)	3 (1.8%)	0 (0%)	3 (4.2%)	**<0**.**001**	**<0**.**001**	**<0**.**001**
IMP-1	41 (16.7%)	41 (52.6%)	14(56.0%)	27 (50.9%)	0 (0%)	0 (0%)	0 (0%)	**<0**.**001**	**<0**.**001**	**<0**.**001**
ICU stay after culture collection^b^	19 (16.0%)	3 (12.5%)	3 (12.5%)	NA	16 (16.8%)	16 (16.8%)	NA	0.761	0.761	NA
Intubation after culture collection^b^	10 (8.3%)	3 (12.5%)	3 (12.5%)	NA	7 (7.4%)	7 (7.4%)	NA	0.420	0.420	NA
Dialysis after culture collection^b^	3 (2.5%)	1 (4.2%)	1 (4.2%)	NA	2 (2.1%)	2 (2.1%)	NA	0.495	0.495	NA
Total length of hospital stay, median (IQR) (day)	61 (26–108)	57 (26–105)	53 (26–100)	61 (18–117)	61 (30–107)	62 (33–101)	61 (24–127)	0.463	0.703	0.565
Total length of hospital stay, median (IQR) (day)^c^	53 (25–96)	50 (22–89)	48 (25–88)	52 (17–100)	53 (26–99)	48 (26–89)	61 (24–120)	0.552	0.839	0.324
Length of hospital stay after culture collection, median (IQR) (day)	30 (13–56)	24 (12–55)	20 (11–56)	24 (11–58)	31 (14–55)	32 (13–55)	30 (16–59)	0.122	0.438	0.194
Length of hospital stay after culture collection, median (IQR) (day)^c^	30 (15–50)	24 (13–45)	29 (17–51)	23 (11–37)	31 (16–53)	32 (15–54)	30 (16–50)	0.133	0.871	0.101
30 Day mortality								0.282	0.470	0.492
Death	31 (12.6%)	13 (16.7%)	5 (20.0%)	8 (15.1%)	18 (10.8%)	12 (12.5%)	6 (8.5%)			
Alive	207 (84.1%)	64 (82.1%)	20 (80.0%)	44 (83.0%)	142 (85.0%)	80 (83.3%)	62 (87.3%)			
Unknown	8 (3.3%)	1 (1.3%)	0 (0%)	1 (1.9%)	7 (4.2%)	4 (4.2%)	3 (4.2%)			

Data are presented as *n* (%) unless indicated otherwise. Bold indicates statistical significance.

ICU, intensive care unit; GES-5, Guiana extended spectrum-5; NA, not available; TMB, tripoli metallo-β-lactamase.

^a^Threshold of significant difference is 0.0167 after Bonferroni correction.

^b^Data were collected only for infected patients. The percentages displayed are the ‘valid percentages,’ i.e. percentages that exclude missing data from the denominator.

^c^Excludes patients who died in hospital (*n* = 60).

### Isolates, carbapenemases and sequence types

A total of 246 isolates were detected, including 78 (31.7%) CRE, 167 (67.9%) CRPA and 1 (0.4%) CRAB. Among the CRE, *Enterobacter hormaechei* [*n* = 20 (25.6%)] was the most common species, followed by *K. pneumoniae* subsp. *pneumoniae* [*n* = 18 (23.1%)] and *Escherichia coli* [*n* = 7 (9.0%)]. In CPE, *K. pneumoniae* subsp. *pneumoniae* was the most common species (Table S2). The respiratory tract was the most common culture site [*n* = 24 (30.8%)], followed by urine [*n* = 22 (28.2%)]. For CRPA, the most common culture site was also the respiratory tract [*n* = 73 (43.7%)], followed by the urinary tract [*n* = 37 (22.2%)], blood [*n* = 16 (9.6%)] and wound [*n* = 15 (9.0%)].

Of the CRE isolates, 74.4% had genes encoding carbapenemase, with 64.1% of those being the *bla*_IMP_ type. *bla*_IMP-1_ [*n* = 41 (52.6%)] was the most common *bla*_IMP_, followed by *bla*_IMP-11_ [*n* = 5 (6.4%)], *bla*_IMP-60_ [*n* = 3 (3.8%)] and *bla*_IMP-6_ [*n* = 1 (1.3%)]. Also, *bla*_NDM-5_ was found in 5.1% (*n* = 4), *bla*_KPC-2_ in 1.3% (*n* = 1), *bla*_OXA-48_ in 1.3% (*n* = 1) and *bla*_GES-24_ in 2.6% (*n* = 2) of the isolates. The most common sequence type (ST) was *E. hormaechei* subsp. *steigerwaltii* ST133 (11.5%), followed by *E. hormaechei* subsp. *hoffmannii* ST78 (9.0%), and *K. pneumoniae* subsp. *pneumoniae* ST12 (5.1%). Seven of the nine isolates (77.8%) of *E. hormaechei* subsp. *steigerwaltii* ST133 and all seven isolates of *E. hormaechei* subsp. *hoffmannii* ST78 carried *bla*_IMP-1_. Among CRPA, genes encoding carbapenemase were detected in only 2.4% of the isolates, including *bla*_IMP-7_ in 1.8% (*n* = 3) and *bla*_GES-5_ in 0.6% (*n* = 1). CRPA was genomically diverse with 98 STs identified, including ST313 (7.2%), ST235 (6.6%) and ST186 (3.6%). All four carbapenemase-producing CRPA isolates were ST235. The only CRAB isolate carried *bla*_OXA-51-like_ and *bla*_TMB-1_ and belonged to ST823, part of the global clone 2. The isolates were therefore diverse across the three pathogens without predominance of specific STs. However, the IMP group carbapenemase gene was often found in CRE regardless of STs.

### Infection and colonization

Based on the study definition, the percentages of infected and colonized patients were 49.2% and 50.8%, respectively. Twenty-five (32.1%) patients with CRE were infected, and 53 (67.9%) were colonized. Bloodstream, respiratory tract and urinary tract infections [*n* = 6 (24.0%)] were the most common among the infected patients, followed by surgical site infection [*n* = 3 (12.0%)]. The bloodstream infection source was secondary in four patients and unknown in two. Regarding CRPA, 96 patients (57.5%) were infected, and 71 (42.5%) were colonized. Respiratory tract infection [*n* = 34 (35.4%)] was the most common, followed by urinary tract infection [*n* = 22 (22.9%)], bloodstream infection [*n* = 16 (16.7%)] and surgical site infection [*n* = 11 (11.5%)] (Table 1). The bloodstream infection source was secondary in 11 patients, central line associated in 3 patients and unknown in 2 patients.

CRPA was thus significantly more likely to cause infection than CRE (*P* = 0.001), with the difference driven by a higher proportion of CRPA respiratory isolates associated with infection.

### Antimicrobial susceptibility

Regarding the CRE isolates, the proportion of MDR isolates was 100%, whereas those of XDR and DTR CRE isolates were 20.5% and 28.2%, respectively (Table 2). Susceptibility rates to amikacin, gentamicin and trimethoprim-sulfamethoxazole were high (91.0%, 92.3% and 76.9% susceptible, respectively).

**Table 2.** Antimicrobial susceptibility of CRE and CRPA isolates

	Isolates susceptible^a^, *n* (%)			*P* value CPE versus non-CPE
Antibiotic	Total (*n* = 78)	CPE (*n* = 58)	Non-CPE (*n* = 20)	
*Enterobacterales*				
Piperacillin-tazobactam	26 (33.3)	23 (39.7)	3 (15.0)	0.056
Cefepime	16 (20.5)	11 (19.0)	5 (25.0)	0.540
Ceftolozane-tazobactam	5 (7.2)	0 (0)	5 (25.0)	**0**.**001**
Imipenem	18 (23.1)	12 (20.7)	6 (30.0)	0.539
Meropenem	18 (23.1)	9 (15.5)	9 (45.0)	**0**.**012**
Aztreonam	32 (41.0)	28 (48.3)	4 (20.0)	**0**.**035**
Ciprofloxacin	23 (29.5)	15 (25.9)	8 (40.0)	0.263
Levofloxacin	21 (26.9)	14 (24.1)	7 (35.0)	0.387
Fosfomycin	45 (57.7)	34 (58.6)	11 (55.0)	0.798
Gentamicin	72 (92.3)	55 (94.8)	17 (85.0)	0.172
Amikacin	71 (91.0)	51 (87.9)	20 (100)	0.181
Colistin^a^	61 (78.2)	51 (87.9)	10 (50.0)	**0**.**001**
Trimethoprim-sulfamethoxazole	60 (76.9)	44 (75.9)	16 (80.0)	1.000
MDR	78 (100)	58 (100)	20 (100)	NA
XDR	16 (20.5)	12 (20.7)	4 (20.0)	1.000
DTR	22 (28.2)	16 (27.6)	6 (30.0)	1.000
*P. aeruginosa*	*n* = 167			
Piperacillin-tazobactam	73 (43.7)			
Ceftazidime	59 (35.3)			
Cefepime	69 (41.3)			
Ceftolozane-tazobactam	107 (79.3)			
Imipenem	14 (8.4)			
Meropenem	26 (15.6)			
Aztreonam	47 (28.1)			
Ciprofloxacin	58 (34.7)			
Levofloxacin	33 (19.8)			
Colistin^a^	146 (87.4)			
Gentamicin	135 (80.8)			
Amikacin	141 (84.4)			
Tobramycin	149 (89.2)			
MDR	152 (91.0)	—	—	—
XDR	116 (69.5)	—	—	—
DTR	42 (25.1)	—	—	—

Isolates for which susceptibility could not be determined were considered non-susceptible. Bold indicates statistical significance.

^a^Since colistin has only intermediate and resistance categories in the CLSI criteria, an MIC ≤ 2 mg/L (intermediate) is indicated as susceptible.

Regarding CRPA, the proportions of MDR, XDR, and DTR isolates were 91.0%, 69.5% and 25.1%, respectively. Amikacin, gentamicin and ceftolozane-tazobactam maintained high rates of activity (84.4%, 80.8% and 79.3% susceptible, respectively). Since CRPA was defined with resistance to either meropenem or imipenem, 15.6% of the isolates were susceptible to meropenem and 8.4% to imipenem. The only CRAB isolate was resistant to ampicillin-sulbactam and cefotaxime but was susceptible to cefepime, ciprofloxacin, levofloxacin and amikacin, thus falling under MDR, but not XDR or DTR.

### Outcomes

Approximately three-quarters (73.1%) of the CRE isolates were detected after Day 6 of hospitalization. The all-cause 30 day mortality rates of all patients with CRE, those with infection and those with colonization were 16.7%, 20.0% and 15.1%, respectively (Table 1). However, the 30 day all-cause mortality rate was as high as 33.3% in patients with bloodstream or respiratory tract infections (Table 3).

**Table 3.** Outcomes of patients with infection due to CRE and CRPA, based on infection types

	CRE	CRPA	
Bloodstream infection^a^	(*n* = 6)	(*n* = 16)	*P* value
Total length of hospital stay, median (IQR) (day)	69 (33–103)	84 (36–141)	0.590
Length of hospital stay after culture collection, median (IQR) (day)	36 (11–78)	31 (21–74)	0.641
30 Day mortality			0.585
Death	2 (33.3%)	3 (18.8%)	
Alive	4 (66.7%)	13 (81.3%)	

Respiratory tract infection^a^	(*n* = 6)	(*n* = 34)	
Total length of hospital stay, median (IQR) (day)	67 (26–99)	62 (34–127)	0.726
Length of hospital stay after culture collection, median (IQR) (day)	24 (6–54)	33 (15–56)	0.343
30 Day mortality			0.703
Death	2 (33.3%)	6 (17.6%)	
Alive	4 (66.7%)	26 (76.5%)	
Unknown	0 (0%)	2 (5.9%)	

Other infection^a^	(*n* = 15)	(*n* = 55)	
Total length of hospital stay, median (IQR) (day)	48 (24–109)	57 (27–98)	0.983
Length of hospital stay after culture collection, median (IQR) (day)	20 (16–49)	35 (12–60)	0.657
30 Day mortality			1.000
Death	1 (6.7%)	3 (5.5%)	
Alive	14 (93.3%)	50 (90.9%)	
Unknown	0 (0%)	2 (3.6%)	

Data are presented as *n* (%) unless indicated otherwise.

^a^Respiratory tract/other infections accompanied by bloodstream infection with the same organism were included for analysis in both groups (CRE, 1 respiratory tract infection, 1 other infection; CRPA, 2 respiratory tract infections, 7 other infections).

Regarding CRPA, 68.3% of the isolates were detected after Day 6 of hospitalization. The all-cause 30 day mortality rates of all patients with CRPA, those with infection and those with colonization were 10.8%, 12.5% and 8.5%, respectively. The 30 day all-cause mortality rate in patients with bloodstream or respiratory tract infections was 18.8% and 17.6%, respectively.

The patient with CRAB was colonized in the bile. The patient had a history of solid organ transplantation, immunosuppressant treatment, chemotherapy and glucocorticoid therapy, but no history of overseas travel. The patient survived through the end of the follow-up period.

### Patients with CRE versus CRPA

**Figure 1.**
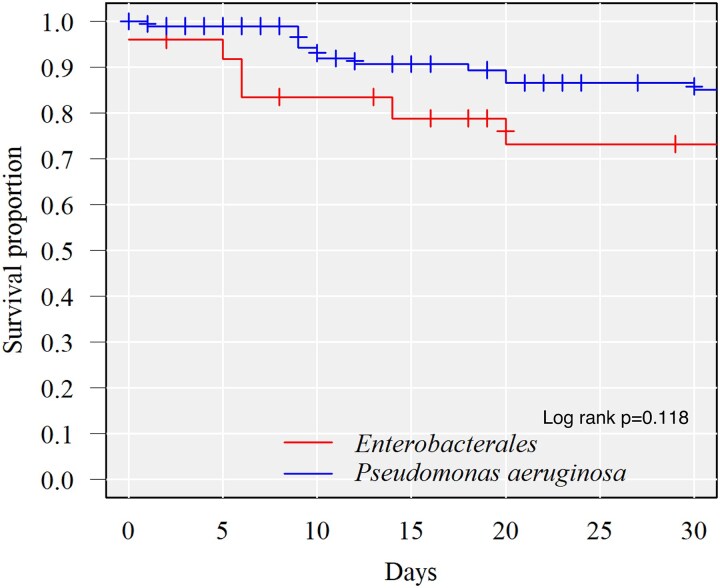
Kaplan–Meier survival curves showing patient days from the first positive culture to death for CRE- and CRPA-infected patients.

When comparing 30 day mortality rates between CRE- and CRPA-infected patients, 30 day mortality rates tended to be higher in CRE-infected patients (20.0% versus 12.5%, no statistically significant difference) (Table 1; Figure 1). The trend remained unchanged after adjusting for background factors using inverse-probability weighting (Table 4; Table S3). In terms of infection type, CRE-infected patients tended to have higher 30 day mortality rates than CRPA-infected patients for bloodstream infections and respiratory tract infections (33.3% versus 18.8%, 33.3% versus 17.6%, respectively).

**Table 4.** Adjusted hazard/risk ratio of clinical outcomes for CRE infection compared with CRPA infection

	Adjusted hazard/risk ratio (95% CI)	*P* value
30 Day mortality^a^	1.71 (0.53–5.55)	0.371
Total length of hospital stay, median (IQR) (day)^b^	0.68 (0.34–1.37)	0.278
Length of hospital stay after culture collection, median (IQR) (day)^b^	0.82 (0.40–1.67)	0.582

CI, confidence interval.

^a^Result of Cox regression analysis.

^b^Results of quasi-Poisson regression analysis.

### Patients with CPE versus non-CPE

The 30 day mortality rates tended to be higher in non-CPE patients compared with CPE patients (20.0% versus 15.5%). Additionally, patients with CPE stayed in the hospital longer after their samples were taken, with an average of 27 days, compared with 17 days for those without CPE (Table S4). The 30 day mortality rates for CPE and non-CPE cases were 18.8% and 22.2% for the infected patients and 14.3% and 18.2% for the colonized patients.

## Discussion

While CRE, CRPA and CRAB each represent a global public health threat, their epidemiology and clinical impact vary substantially across regions and countries. The present study was carried out to clarify the clinical characteristics and outcomes of patients hospitalized with CRE, CRPA and CRAB in Japan. We also determined traits of the bacterial isolates, such as their resistance to antibiotics, STs and carbapenemase genes, to gain insights into the contemporary epidemiology of the three critical carbapenem-resistant pathogens in the country.

In this study, CRPA accounted for the largest proportion; however, the 30 day mortality rate in infected patients tended to be higher with CRE than with CRPA (20.0% versus 12.5%). In patients with CRE, however, the 30 day mortality rate of patients with non-CPE was numerically higher than that of patients with CPE (20.0% versus 15.5%) (Table S4). A previous report by Tamma *et al.*^27^ showed that the 30 day mortality rate of CPE infection mostly producing KPC carbapenemase was four times higher than that of non-CPE, which differs from the results of the present study. Although CPE receives special attention as a high-priority threat, our findings suggest that non-CPE CRE also should not be underestimated and need to be closely monitored.

The 30 day mortality rates of CPE cases were 18.8% for the infection cases and 14.3% for the colonization cases. This is consistent with previous studies reporting 30 day mortality rates of 16%–27% for IMP-producing CPE infection.^5,6^ The relatively small difference may be explained by the high proportion of respiratory isolates among the colonization cases. Howard-Anderson *et al.*^28^ reported no difference in mortality between CRE-infected and colonized cases. However, they did find that the location of the bacteria was associated with mortality, with higher mortality rates in both infected and colonized cases when the isolates were from the respiratory tract.

A majority of the CRE isolates produced IMP metallo-β-lactamase, whereas carbapenemase production in CRPA was rare. Among the IMP-type carbapenemases, IMP-1 was the most common, followed by IMP-11, IMP-60 and IMP-6. In Japan, the predominant carbapenemase is IMP-1, in contrast to China and Taiwan, which primarily report IMP-4 and IMP-8, respectively.^29–31^ In contrast, the globally predominant carbapenemases NDM and KPC were only detected in five cases. Four of these five patients had a history of overseas travel within 3 months, suggesting that they represented imported cases.

In the CPE isolates, the percentage of XDR reached 100%, while the percentage of DTR was only 27.6%. This finding is similar to a previous report in which IMP-producing CPE was less susceptible to β-lactams, such as piperacillin, piperacillin-tazobactam, and cefepime.^6^ Although susceptibility of CPE to aminoglycosides was also maintained, future trends need to be monitored especially in view of the recent revision of the breakpoints by the CLSI.^32^ Conventional treatment options such as aminoglycosides and trimethoprim-sulfamethoxazole are available for some CRE infection cases in Japan, which may explain the low mortality rate observed in the study.

The 30 day mortality rates of CRPA were unexpectedly low at 12.5% in infected patients and 8.5% in colonized patients. Previous studies on CRPA have reported mortality rates as high as 19%–35% for bacteraemia, 19% for respiratory tract infections and 7% for urinary tract infections.^33,34^ In this study, the 30 day mortality rates were 18.8% for bloodstream infections, 17.6% for respiratory tract infections and 0% for urinary tract infections, respectively. The lower mortality rate in the present study may be related to the relatively low proportion of bloodstream infections (16.7%) and the high proportion of urinary tract infections accounting for 21.9% of the infection cases. The low 30 day mortality rate may also have been influenced by the fact that only 25.1% of the isolates were DTR and many isolates remained susceptible to at least some antipseudomonal β-lactams or fluoroquinolones.

CRAB accounted for only 0.4% of the total isolates in our study. Although the carbapenem resistance rate of *A. baumannii* is reported to be 40% in North America, 47% in Europe and 50%–92% in Southeast Asia, the rate is <5% in Japan.^11,14,35^ According to the study by Matsui *et al.*,^36^ the low rate of carbapenem resistance may be related to the scarcity of the MDR global clone 2 and the presence of sporadic, non-MDR STs in Japan. In Australia, which, like Japan, is geographically isolated, low rates of CRAB have also been reported, suggesting that a limited influx of persons with nosocomial exposure from outside the countries may help explain the low incidence of CRAB.^37^

Determining whether isolates detected from non-sterile sites represent infection or colonization is often challenging. In this study, 32.1% of CRE cases were classified as infections, while 67.9% were classified as representing colonization. These proportions align closely with previous reports from Japan, where 37.5% of cases were categorized as infections.^5^ To enhance rigour of our data, we implemented pre-defined diagnostic criteria across the study sites to minimize variation among researchers and excluded isolates obtained for surveillance purposes.^19–22^

Our study has several limitations. First, the distinction between infected and colonized patients was a challenge. We suspect that some infected patients may have been misclassified as colonized due to the rigorous definitions of infection. However, when standardized definitions are employed, the likelihood of misclassifying colonized patients as infected patients is considered to be low. Second, we only collected specimens from several regions in the country. However, the participating facilities were distributed across the country. Third, treatment factors, such as medications and time to appropriate therapy, were not included in the propensity score adjustment because of the variety of treatments and the difficulty of correction. Fourth, because of the restricted number of CRE patients, comparisons of CPE and non-CPE were made for infected and colonized patients combined. Finally, the data represent a snapshot of the epidemiology of CRGNR, which may be evolving continuously.

### Conclusions

Our study revealed the unique molecular and clinical epidemiology of CRGNR in Japan, which will inform strategies for diagnosis and treatment of infected patients.

## Supplementary Material

dlaf027_Supplementary_Data

## Supplementary data

Tables S1–S4 are available as Supplementary data at *JAC-AMR* Online.
