# Supplementary material for: Molecular epidemiology and patient outcome of carbapenem-resistant Enterobacterales, Pseudomonas aeruginosa and Acinetobacter baumannii in Japan: a multicenter study from MultiDrug-Resistant organisms clinical research network
Source: JAC Antimicrob Resist. 2025 Mar 6;7(2):dlaf027. doi: 10.1093/jacamr/dlaf027 (PMC11882498; doi:10.1093/jacamr/dlaf027)
Supplement: dlaf027_Supplementary_Data [file dlaf027_supplementary_data.docx]

**Supplemental Materials**

**Table S1.** Reference genomes for *Enterobacterales.*

| **Organism** | **Taxonomy id** | **RefSeq_Assembly Accession** | **BioProject** | **BioSample** | **Strain** |
| --- | --- | --- | --- | --- | --- |
| *Citrobacter braakii* | 57706 | GCF_009648935.1 | PRJNA541977 | SAMN10574724 | MiY-A |
| *Citrobacter freundii* | 546 | GCF_003812345.1 | PRJNA231221 | SAMN10163242 | FDAARGOS_549 |
| *Citrobacter koseri* | 290338 | GCF_000018045.1 | PRJNA12716 | SAMN02603912 | ATCC BAA-895 |
| *Enterobacter asburiae* | 61645 | GCF_001521715.1 | PRJNA285282 | SAMN03742638 | ATCC 35953 |
| *Enterobacter cloacae* (*Enterobacter cloacae* subsp. *cloacae*) | 716541 | GCF_000025565.1 | PRJNA45793 | SAMN02603901 | ATCC 13047 |
| *Enterobacter hormaechei* | 158836 | GCF_019048625.1 | PRJNA231221 | SAMN16357577 | FDAARGOS 1435 |
| *Enterobacter kobei* | 208224 | GCF_018323985.1 | PRJDB11476 | SAMD00294602 | JCM 8580 |
| *Enterobacter quasiroggenkampii* | 2497436 | GCF_003964805.1 | PRJNA415108 | SAMN10525003 | 90040 |
| *Enterobacter roggenkampii* | 1812935 | GCF_001729805.1 | PRJNA259658 | SAMN05581750 | DSM 16690 |
| *Escherichia coli* | 511145 | GCF_000005845.2 | PRJNA225 | SAMN02604091 | K-12 substr. MG1655 |
| *Klebsiella aerogenes* | 548 | GCF_007632255.1 | PRJNA555744 | SAMN12330869 | Ka37751 |
| *Klebsiella michiganensis* | 1134687 | GCF_015139575.1 | PRJDB9036 | SAMD00196009 | THO-011 |
| *Klebsiella oxytoca* | 571 | GCF_003812925.1 | PRJNA231221 | SAMN10163176 | FDAARGOS_500 |
| *Klebsiella pneumoniae* (*Klebsiella pneumoniae* subsp. *pneumoniae*) | 1125630 | GCF_000240185.1 | PRJNA78789 | SAMN02602959 | HS11286 |
| *Klebsiella variicola* | 244366 | GCF_009648975.1 | PRJNA541977 | SAMN10574723 | LEMB11 |
| *Proteus mirabilis* | 529507 | GCF_000069965.1 | PRJNA12624 | SAMEA1705945 | HI4320 |
| *Serratia marcescens* | 615 | GCF_003516165.1 | PRJNA438529 | SAMN08720168 | KS10 |

**Table S2.** Isolated species of CPE and non-CPE

|  | CPE  (n = 58) | Non-CPE  (n = 20) |
| --- | --- | --- |
| *Citrobacter braakii* | 0 (0%) | 1 (5.0%) |
| *Citrobacter freundii* | 4 (6.9%) | 0 (0%) |
| *Citrobacter koseri* | 2 (3.4%) | 1 (5.0%) |
| *Enterobacter asburiae* | 3 (5.2%) | 1 (5.0%) |
| *Enterobacter cloacae* | 1 (1.7%) | 0 (0%) |
| *Enterobacter hormaechei subsp. hoffmannii* | 7 (12.1%) | 0 (0%) |
| *Enterobacter hormaechei subsp. steigerwaltii* | 11 (19.0%) | 2 (10.0%) |
| *Enterobacter kobei* | 3 (5.2%) | 1 (5.0%) |
| *Enterobacter quasiroggenkampii* | 0 (0%) | 1 (5.0%) |
| *Enterobacter roggenkampii* | 0 (0%) | 1 (5.0%) |
| *Escherichia coli* | 6 (10.3%) | 1 (5.0%) |
| *Klebsiella aerogenes* | 0 (0%) | 3 (15.0%) |
| *Klebsiella michiganensis* | 3 (5.2%) | 0 (0%) |
| *Klebsiella oxytoca* | 0 (0%) | 1 (5.0%) |
| *Klebsiella pneumoniae subsp. pneumoniae* | 16 (27.6%) | 2 (10.0%) |
| *Klebsiella variicola* | 0 (0%) | 1 (5.0%) |
| *Proteus mirabilis* | 1 (1.7%) | 2 (10.0%) |
| *Serratia marcescens* | 1 (1.7%) | 2 (10.0%) |

Abbreviations: CPE, carbapenemase-producing *Enterobacterales*.

**Table S3.** Inverse probability weight adjusted patient characteristics

|  | **CRE**  **(n = 17.8)** | **CRPA**  **(n = 95.0)** | **Standardized mean difference** |
| --- | --- | --- | --- |
| **Age [mean, SD] (year)** | 66.4 [18.6] | 66.3 [19.3] | 0.105 |
| **Sex [%] (Male)** | 66.3 | 55.8 | 0.060 |
| **Charlson’s combined condition score [mean, SD]** | 3.0 [2.5] | 2.5 [2.2] | 0.190 |
| **Pitt bacteremia score [mean, SD]** | 1.6 [2.9] | 1.9 [2.1] | 0.102 |
| **Bloodstream infection [%]** | 16.1 | 18.1 | 0.019 |
| **Respiratory tract infection [%]** | 37.4 | 33.1 | 0.043 |

Abbreviations: CRE, carbapenem-resistant *Enterobacterales*; CRPA, carbapenem-resistant *Pseudomonas* aeruginosa; SD, standard deviation; IQR, interquartile range.

**Table S4.** Demographic characteristics and clinical outcomes of patients with CPE and non-CPE.

|  | **All**  **(n = 78)** | **CPE**  **(n = 58)** | **Non-CPE**  **(n = 20)** | ***P* value** |
| --- | --- | --- | --- | --- |
| **Age [median, IQR] (year)** | 70 [64–79] | 71 [58–79] | 69 [65–79] | 0.986 |
| **Sex (Male)** | 51 (65.4%) | 38 (65.5%) | 13 (65.0%) | 1.000 |
| **From** |  |  |  | 0.150 |
| **Home** | 55 (70.5%) | 39 (67.2%) | 16 (80.0%) |  |
| **Another hospital** | 15 (19.2%) | 14 (24.1%) | 1 (5.0%) |  |
| **Nursing home** | 7 (9.0%) | 4 (6.9%) | 3 (15.0%) |  |
| **Birth** | 1 (1.3%) | 1 (1.7%) | 0 (0%) |  |
| **Acute and chronic conditions on admission** |  |  |  |  |
| **Myocardial infarction** | 3 (3.8%) | 3 (5.2%) | 0 (0%) | 0.565 |
| **Congestive heart failure** | 10 (12.8%) | 7 (12.1%) | 3 (15.0％) | 0.711 |
| **Peripheral vascular disease** | 3 (3.8%) | 1 (1.7%) | 2 (10.0%) | 0.160 |
| **Cerebrovascular disease** | 14 (17.9%) | 10 (17.2%) | 4 (20.0%) | 0.746 |
| **Dementia** | 7 (9.0%) | 6 (10.3%) | 1 (5.0%) | 0.670 |
| **Chronic obstructive**  **pulmonary disease** | 1 (1.3%) | 1 (1.7%) | 0 (0%) | 1.000 |
| **Connective tissue disease** | 1 (1.3%) | 1 (1.7%) | 0 (0%) | 1.000 |
| **Diabetes mellitus** | 20 (25.6%) | 17 (29.3%) | 3 (15.0%) | 0.249 |
| **Any liver disease** | 5 (6.4%) | 3 (5.2%) | 2 (10.0%) | 0.598 |
| **Any renal diseases** | 7 (9.0%) | 5 (8.6%) | 2 (10.0%) | 1.000 |
| **Hemiplegia** | 2 (2.6%) | 1 (1.7%) | 1 (5.0%) | 0.450 |
| **Leukemia** | 1 (1.3%) | 0 (0%) | 1 (5.0%) | 0.256 |
| **Localized solid tumor** | 13 (16.7%) | 8 (13.8%) | 5 (25.0%) | 0.300 |
| **Metastatic solid tumor** | 4 (5.1%) | 1 (1.7%) | 3 (15.0%) | 0.050 |
| **Charlson’s combined condition score [median, IQR]** | 1 [0–3] | 1 [0–3] | 2 [1–4] | 0.063 |
| **Immunosuppressive state** |  |  |  |  |
| **Glucocorticoid therapy** | 11 (14.1%) | 9 (15.5%) | 2 (10.0%) | 0.719 |
| **Antineoplastic chemotherapy** | 3 (3.8%) | 2 (3.4%) | 1 (5.0%) | 1.000 |
| **Biological agents** | 3 (3.8%) | 2 (3.4%) | 1 (5.0%) | 1.000 |
| **Immunosuppressant** | 3 (3.8%) | 2 (3.4%) | 1 (5.0%) | 1.000 |
| **Solid organ transplantation** | 1 (1.3%) | 0 (0%) | 1 (5.0%) | 0.256 |
| **Overseas travel history in the past 3 month** |  |  |  | **0.012** |
| **Yes** | 6 (7.7%) | 5 (8.6%) | 1 (5.0%) |  |
| **No** | 56 (71.8%) | 37 (63.8%) | 19 (95.0%) |  |
| **Unknown** | 16 (20.5%) | 16 (27.6%) | 0 (0%) |  |
| **Location at time of culture collection** |  |  |  | 0.830 |
| **Emergency department** | 2 (2.6%) | 2 (3.4%) | 0 (0%) |  |
| **ICU** | 9 (11.5%) | 6 (10.3%) | 3 (15.0%) |  |
| **Ward** | 67 (85.9%) | 50 (86.2%) | 17 (85.0%) |  |
| **Pitt bacteremia score [median, IQR]** | 1 [0–4] | 2 [1–4] | 0 [0–1] | **< 0.001** |
| **Duration from hospitalization to culture collection** |  |  |  | 0.172 |
| **the same day** | 11 (14.1%) | 9 (15.5%) | 2 (10.0%) |  |
| **On days 1–5** | 10 (12.8%) | 5 (8.6%) | 5 (25.0%) |  |
| **After day 6** | 57 (73.1%) | 44 (75.9%) | 13 (65.0%) |  |
| **Isolation sites** |  |  |  |  |
| **Blood** | 6 (7.7%) | 5 (8.6%) | 1 (5.0%) | 1.000 |
| **Respiratory** | 24 (30.8%) | 20 (34.5%) | 4 (20.0%) | 0.273 |
| **Urine** | 22 (28.2%) | 18 (31.0%) | 4 (20.0%) | 0.402 |
| **Wound** | 3 (3.8%) | 2 (3.4%) | 1 (5.0%) | 1.000 |
| **Stool** | 12 (15.4%) | 8 (13.8%) | 4 (20.0%) | 0.492 |
| **Other** | 13 (16.7%) | 7 (12.1%) | 6 (30.0%) | 0.084 |
| **Infection** | 25 (32.1%) | 16 (27.6%) | 9 (45.0%) | 0.050 |
| **Type of infection** |  |  |  |  |
| **Bloodstream infection** | 6 (7.7%) | 5 (8.6%) | 1 (5.0%) | 1.000 |
| **Respiratory tract infection** | 6 (7.7%) | 5 (8.6%) | 1 (5.0%) | 1.000 |
| **Urinary tract infection** | 6 (7.7%) | 5 (8.6%) | 1 (5.0%) | 1.000 |
| **Surgical site infection** | 3 (3.8%) | 1 (1.7%) | 2 (10.0%) | 0.160 |
| **Others** | 6 (7.7%) | 2 (3.4%) | 4 (20.0) | **0.035** |
| **ICU stay after culture collection** ^a^ | 4 (16.0%) | 3 (18.8%) | 1 (11.1%) | 1.000 |
| **Intubation after culture collection** ^a^ | 3 (12.0%) | 3 (18.8%) | 0 (0%) | 0.280 |
| **Dialysis after culture collection** ^a^ | 1 (4.0%) | 1 (6.3%) | 0 (0%) | 1.000 |
| **Total length of hospital stay [median, IQR] (day)** | 57 [26–105] | 72 [26–117] | 39 [18–80] | 0.054 |
| **Total length of hospital stay [median, IQR] (day)** ^b^ | 50 [22–89] | 64 [25–112] | 39 [18–80] | 0.198 |
| **Length of hospital stay after culture collection [median, IQR] (day)** | 24 [12–55] | 28 [15-72] | 14 [4–28] | **0.013** |
| **Length of hospital stay after culture collection [median, IQR] (day)** ^b^ | 24 [13–45] | 27 [15–57] | 17 [6–34] | 0.144 |
| **30-day mortality** |  |  |  | 0.801 |
| **Death** | 13 (16.7%) | 9 (15.5%) | 4 (20.0%) |  |
| **Alive** | 64 (82.1%) | 48 (82.8%) | 16 (80.0%) |  |
| **Unknown** | 1 (1.3%) | 1 (1.7%) | 0 (0%) |  |

Abbreviations: CPE, carbapenemase-producing *Enterobacterales*; IQR, interquartile range; ICU, intensive care unit.

Data are presented as n (%) unless indicated otherwise.

^a^Data were collected only for infected patients. The percentages displayed are the “valid percentages,” excluding missing data from the denominator.

^b^Excludes patients who died in hospital (n=24).
